# Study on the effect of different types of sugar on proliferation and inflammatory in goose fatty liver

**DOI:** 10.3389/fvets.2025.1625050

**Published:** 2025-11-25

**Authors:** Shuang Yi, Yongqiang Teng, Li Zhou, Jiang Li, Shanjing Peng, Shouhai Wei, Chunchun Han

**Affiliations:** 1 Farm Animal Germplasm Resources and Biotech Breeding Key Laboratory of Sichuan Province, College of Animal Science and Technology, Sichuan Agricultural University, Chengdu, China; 2 Yibin Academy of Agricultural Sciences, Yibin, China

**Keywords:** glucose, fructose, proliferation, inflammation, goose fatty liver

## Abstract

This study aimed to investigate the regulatory effects of dietary sugar types on hepatocyte proliferation and inflammatory cytokine expression during fatty liver formation in geese. One hundred geese were randomly divided into five groups: control group, corn flour group, glucose group, fructose group, and sucrose group, receiving force-feeding for 21 days. Primary hepatocytes isolated from 21-day-old geese were treated with 30 mmol/L glucose or fructose, combined with CPT1A gene interference. Fructose significantly enhanced lipid accumulation in overfed geese (*p* < 0.05). Hepatic transcriptome analysis revealed that dietary 10% glucose upregulated differentially expressed genes involved in cell growth and proliferation, with carnitine palmitoyltransferase 1A (CPT1A) being the most noteworthy candidate. Glucose treatment upregulated *CyclinD1* and *CyclinD2* expression and promoted hepatocyte proliferation, while fructose increased *p21* and *p27* expression (*p* < 0.05). Fructose reduced *TNF-α* and *IL-6* expression, whereas glucose elevated IL-6 levels (*p* < 0.05). Following *CPT1A* interference, *CyclinD1* and *CyclinD3* expression increased in primary hepatocytes. Glucose combined with si-*CPT1A* treatment decreased *CyclinD3* while increasing *p21* expression. Both glucose and fructose synergistically with si-*CPT1A* reduced *IL-6* expression (*p* < 0.05). In conclusion, glucose promotes the proliferation of goose hepatocytes by activating cell cycle genes and modulates the interaction between lipid metabolism and inflammation, whereas fructose regulates inflammatory signaling to induce controlled inflammatory responses and enhance fat deposition.

## 1 Introduction

As an economically important poultry species, geese provide valuable products such as down feathers, meat, and fatty liver (foie gras), the latter of which is highly prized in gourmet markets due to its unique nutritional profile and flavor. The formation of goose fatty liver involves a disruption in the balance between hepatic lipid synthesis, transport, and fatty acid β-oxidation, ultimately leading to abnormal triglyceride accumulation in hepatocytes (1). This process is closely associated with insulin resistance (IR), endoplasmic reticulum stress (ERS), and hepatocyte growth and proliferation (2). In recent years, goose fatty liver has been recognized as an ideal model for studying non-alcoholic fatty liver disease (NAFLD) (3), the most prevalent chronic liver disorder globally. NAFLD progresses from simple steatosis to non-alcoholic steatohepatitis (NASH, characterized by ballooning degeneration and inflammatory infiltration), fibrosis, and even hepatocellular carcinoma (4, 5). An international expert consensus redefined NAFLD as metabolic dysfunction-associated steatosis liver disease (MASLD) to better reflect its association with metabolic disorders (6). It is worth noting that there are significant species differences between the goose fatty liver model and the mammalian NAFLD model. Although goose fatty liver exhibits severe steatosis (7), it lacks the pronounced inflammation, fibrosis, and pathological changes typically observed in conditions such as fatty liver hemorrhagic syndrome in laying hens or metabolic dysfunction-associated fatty liver disease (MAFLD) in mice (8, 9). Remarkably, cessation of force-feeding leads to the reversible restoration of normal liver structure, suggesting that geese possess specific protective mechanisms that enable their livers to tolerate the lipotoxic effects of extreme steatosis (10). Among these mechanisms, the suppression of hepatic inflammatory responses appears critical, allowing the liver to maintain basic functionality despite massive lipid deposition and creating a favorable microenvironment for fatty liver development (7). In mammalian NAFLD progression, pro-inflammatory factors such as TNF-α and IL-6 typically exacerbate liver damage by activating inflammatory pathways (11, 12). Intriguingly, however, these cytokines have been shown to promote proliferation in certain pathological contexts, such as fibroblast-like synovial cells of rheumatoid arthritis (13).

In goose fatty liver production, force-feeding with high-energy diets is the primary method to induce hepatic lipid deposition. Studies have demonstrated that overfeeding significantly alters hepatic inflammatory responses and cell proliferation. For instance, force-fed Pekin ducks exhibit downregulation of inflammation- and immune-related genes alongside upregulated proliferation-associated genes (14). Similarly, overfed mice develop hepatic steatosis and inflammation, with fructose or glucose supplementation further aggravating liver injury (15). Different carbohydrates (glucose, fructose, and sucrose) play distinct roles in lipogenesis, with notable metabolic differences. Glucose serves as a universal energy substrate utilized by all tissues, whereas fructose is predominantly metabolized in the liver, bypassing rate-limiting enzymes and insulin regulation. Excessive fructose intake leads to fructose-1-phosphate accumulation, ATP depletion, and increased production of uric acid and lactate (16).

Our previous studies have elucidated the effects of different sugars on goose fatty liver formation through the gut-liver axis, endoplasmic reticulum stress, and lipid deposition pathways, revealing that glucose and fructose supplementation upregulate cell cycle and DNA replication pathways while downregulating pro-inflammatory genes (17–19). Nevertheless, the precise mechanisms by which sugars modulate inflammatory responses and hepatocyte proliferation during fatty liver development remain unclear and warrant systematic investigation. Based on this, this study aims to investigate the impact of dietary sugar types on hepatocyte proliferation and inflammatory cytokine expression in goose fatty liver through both hepatic transcriptome analysis and primary hepatocyte experiments.

## 2 Materials and methods

### 2.1 Animals and sample collection

A total of 100 healthy male Tianfu meat geese aged 80 d with similar body weight were provided by the waterfowl breeding unit of Sichuan Agricultural University (Ya’an, China). These selected geese were randomly divided into a control group (*n* = 20) and four different overfeeding groups (*n* = 20 in each group), including corn flour group, glucose group, fructose group and sucrose group. During the experiment, the control group of geese were allowed to have free access to corn flour and water, with an average daily consumption of approximately 300 grams. The ganders of control group were fed maize flour (dry matter: water = 1:1). In the four overfeeding groups, geese were overfed with a carbohydrate-rich diet, which was composed of cooked corn flour supplemented with 2% fish meal, either 2.5% or 2% soybean oil, and 1% salt. The daily intake for each overfeeding group was set at 1600 g. Three different types of sugar were incorporated into the overfed diet with, respectively, 10% glucose, 10% fructose, or 10% sucrose by weight of the diet. Additionally, there was one overfeeding group overfed with diet without supplementary sugar. The detailed feeding protocols and procedures were conducted in compliance with the relevant diets regimes previously reported (2). The goose is secured in a poultry holder. One feeder controls the goose’s head with one hand, fixing the base of the beak, and uses the other hand to reach into the mouth to press the base of the tongue, thereby prying open the beak. Another feeder applies a small amount of grease to the feeding tube for lubrication, then gently inserts it into the goose’s beak, guiding it along the pharynx and esophagus to the anterior part of the esophagus. After insertion, the feeder steps on the feeding switch to activate the pressure pusher, which propels the feed into the esophagus. Meanwhile, the left hand keeps the head fixed, and the right hand feels the expanded part of the esophagus. Once the esophagus is full, the switch is released to stop the feed delivery. The feeding tube is slowly withdrawn, and finally, the head is released, and the goose is gently returned to the pen.

To facilitate experimental geese to adapt to forced feeding, the geese of the overfeeding groups were subjected to a 14d pre-feeding period, during which the daily feed intake was gradually increased to 1,600 g. Subsequently, there was a 21d overfeeding period, during which the geese were overfed with four meals a day with water ad libitum. At the end of the overfeeding period, all geese were weighed following a 12 h overnight fast with water provided. Approximately 10 mL of blood is drawn from the wing veins of the selected geese (*n* = 6). After centrifugation, the obtained serum was stored at −20 °C for subsequent analysis. The experimental geese were killed with an electrolethaler at 16 weeks of age to obtain liver samples. The liver weight of each goose was measured and recorded. A small piece of liver tissue was collected from each goose immediately. The samples were rapidly frozen in liquid nitrogen and stored at −80 °C for follow-up experiments.

### 2.2 Transcriptome sequencing and analysis

In this study, 15 geese representing three biological replicates for each group (control group, corn flour group, glucose group, fructose group, and sucrose group) were randomly selected (*n* = 3). Total RNA was extracted from approximately 100 mg of frozen liver tissue from each trial group using the RNeasy Mini Kit (QIAGEN, Germany), strictly following the manufacturer’s instructions. The purity and integrity of the RNA were evaluated using the Agilent Bioanalyzer 2,100 (Agilent Technologies, Shanghai, China). Liver RNA samples, each containing approximately 1 μg of RNA from different individuals, were sent to Bemike (Beijing, China) for the construction of a double-ended sequencing library. The sequencing libraries were generated using the NEBNext Ultra™ RNA Library Prep Kit for Illumina NexSeq500 (NEB, USA) according to the manufacturer’s protocol, with index codes added to the attribute sequences of each sample. All libraries were sequenced on the Illumina NexSeq500 platform with a read length of 300 bp (Baimike biological Technology Co., LTD, Beijing, China).

Clean reads were mapped to the geese reference genome (AnsCyg_PRJNA183603_v1.0) by Toptat2 with default parameters. The number of aligned reads per gene was calculated using reads per kilobase of transcript per million fragments mapped (RPKM). The DEGseq method was used for differential expression analysis among the five groups, with a *p-value* <0.05. The statistical enrichment of differentially expressed genes (DEGs) in KEGG pathways was assessed by the KOBAS software. Transcriptome analysis was conducted via the Baimike BioCloud platform. The DEGs identified were subjected to functional annotation. The DEGs data utilized in this study were uploaded to the STRING database (https://cn.string-db.org/) to construct a protein–protein interaction network.

### 2.3 Serum parameter detection

The assay kits that detected Triglyceride (TG), total protein (TP), immunoglobulin A (IgA), albumin (ALB), interleukin 2 (IL*-*2), C-reactive protein (CRP), alanine aminotransferase (ALT) and aspartate aminotransferase (AST) were provided by Nanjing Jiancheng Bioengineering Institute (Nanjing, China). The content of TP, IgA, ALB, IL*-*2, ALT and AST were detected by ELISA. For the detection of CRP using emulsion-enhanced immunoturbidimetry, the method is based on the principle that latex particles coated with specific antibodies can specifically bind to the target antigen in the serum. All operation steps strictly follow the instructions of the reagent kit and all assays were carried out in triplicate.

### 2.4 Long-chain fatty acid of foie Gras determination

A 0.2 g sample of foie gras (accurately weighed to 0.0001 g) was mixed with 0.5 mL of 0.5 g/L glyceryl trinundecanoate (internal standard, Sigma, USA), 2 mL of 95% ethanol, 2 mL of water, 0.1 g of pyrogallic acid (accurately weighed to 0.0001 g), and 10 mL of 8.3 mol/L HCl in a 50 mL conical flask. The flask was sealed and hydrolyzed at 80 °C for 40 min with shaking every 10 min. After cooling, the hydrolysate was transferred to a 250 mL separatory funnel, and the flask was rinsed with 10 mL of 95% ethanol. Lipids were extracted by adding 50 mL of ether/petroleum ether (1,1, v/v), shaking for 5 min, and allowing phase separation. The upper organic layer was collected, and the extraction was repeated twice. The combined extracts were concentrated using a rotary evaporator at 55 °C. Subsequently, saponification and fatty acid methylation are carried out. The lipid residue was saponified with 8 mL of 2% NaOH-methanol at 80 °C for 30 min. Then, 7 mL of 15% BF₃-methanol was added for methylation (30 min). After cooling, 10 mL of n-heptane was added, followed by saturated NaCl solution. The n-heptane layer was dehydrated with anhydrous Na₂SO₄, filtered (0.22 μm), and analyzed. Finally, GC-FID Analysis was conducted. Separation was performed using a temperature program: 130 °C (5 min), then 4 °C/min to 240 °C (30 min). Carrier gas flow: 0.5 mL/min; split ratio: 10:1; injector/detector (FID) temperatures: 250 °C. Fatty acid standards and samples were analyzed under identical conditions.

### 2.5 Isolation and treatment of goose primary hepatocytes

Primary hepatocytes were isolated from the livers of three 14-day-old male Tianfu geese using the two-step collagenase perfusion method described by Seglen. Particularly, the procedure in this study was conducted on livers that had been removed from the geese. Cells were seeded into culture plates at a density of 3 × 10^5^ cells per well and then transferred to an incubator set at 37 °C with 5% CO₂ for cultivation. After 3 h of culture, the cells were washed with phosphate-buffered saline (PBS) to remove non-adherent cells, and the culture medium was replaced with DMEM containing 10% fetal bovine serum (FBS) and 1% penicillin–streptomycin for continued incubation. After 24 h of incubation, the cells were treated with DMEM medium supplemented with either 30 mmol/L glucose or 30 mmol/L fructose and incubated for an additional 24 h. The present study used 30 mmol/L glucose and fructose to treat goose primary hepatocytes (18), as this concentration was confirmed by our preliminary experiments to effectively induce lipid deposition in hepatocytes and falls within the commonly reported concentration range 5–100 mmol/L for avian hepatocyte studies (20, 21). Cells in the control group were maintained in DMEM medium without added sugars. After 24 h, all hepatocytes were harvested for further use.

### 2.6 CPT1A knockdown in goose primary hepatocytes

For *CPT1A* knockdown, and siRNA (si*CPT1A*) targeting *CPT1A* (sense: 5’-CATGCCATCTTGCTCTACCG-3′, antisense: 5’-CAGGGC AAGTTGAACG-AAGG-3′) and a negative control (sense: 5′- GGCTCTGAGCGTGTCCTGA-3′, antisense: 5’-CTGGAACCGGC AATCGTG-3′) were sourced from Baimike biological Technology Co., LTD (Beijing, China). For transfection, Lipofectamine liposomal transfection reagent (Biyun Tian, Shanghai, China) was employed according to the manufacturer’s guidelines. Hepatocytes were treated with si*CPT1A* in culture media supplemented with 30 mmol/L glucose or 30 mmol/L fructose, respectively. After 24 h of co-treatment with si*-CPT1A* and sugar, the cells were collected for Trizol reagent to prepare for total cellular RNA extraction.

### 2.7 Concentration measurement of TNF-*α* and IL-6 in goose primary hepatocytes

After treating the cultured cells with glucose or fructose, the culture media were collected to determine the extracellular concentrations of IL*-*6 and TNF*-*α. Additionally, cell samples were collected for measuring the intracellular concentrations of these cytokines. Briefly, the cell suspensions were diluted with PBS (pH 7.2–7.4) to achieve a concentration of approximately 1 million cells/mL. The cells were lysed by repeated freezing and thawing to release their contents. The supernatant was carefully collected after centrifugation at 2000–3000 rpm for 20 min. ELISA kits for the detection of IL-6 and TNF-*α* were obtained from Nanjing Jiancheng Bioengineering Institute (Nanjing, China). Measurements were performed in accordance with the manufacturer’s instructions. All assays were performed in triplicate.

### 2.8 EdU cell proliferation assay

The proliferation of hepatocytes was assessed using the Cell Light™ EdU kit (RiboBio, Guangzhou, China) following the manufacturer’s protocol. The number of 5-ethynyl-2′-deoxyuridine (EdU)-positive cells was quantified using a fluorescent microscope.

### 2.9 Quantitative real-time PCR

Total RNA was extracted from goose primary hepatocytes used in cell experiment according to the instructions for Trizol reagent. The RNA integrity was assessed using the Agilent 2,100 Bioanalyzer (Agilent Technologies, CA, USA). And cDNA was synthesized for Real-Time PCR according to the instructions of the PrimeScript RT reagent kit (Takara, Dalian, China). Primers were designed through the online software provided by NCBI and were shown in Table 1. Actin β (β-actin) or glyceraldehyde-3-phosphate dehydrogenase (GAPDH) was designated as the internal reference gene for quantitative real-time PCR. Each 25 μL reaction volume contained 12.5 μL of SYBR Premix Ex Taq (2×), 0.5 μL of each forward and reverse primer, 2.0 μL of cDNA template, and ddH₂O was added to adjust the final volume. PCR reaction procedure comprised predevaluation at 95 °C for 10 min, denaturation at 95 °C for 30 s, annealing at 62 °C for 1 min, 40 cycles. Each sample was repeated 3 times. The mRNA expression levels of target genes were calculated using the 2^-ΔΔCt^ method (22).

**Table 1:** Primers for qRT-PCR.

Gene name	Primer sequence (5′ - 3′)		Size (bp)
β-actin	F: CAACGAGCGGTTCAGGTGT	R: TGGAGTTGAAGGTGGTCTCG	92
GAPDH	F: TGAAAGTCGGAGTCAACGGA	R: ACCACTTGGACTTTGCCAGA	82
TNF-α	F: GCAGAGATGGGGATTGTCTTCA	R: CACCAAAGCAAGCTGATGGC	261
IL-6	F: AGCCTCACCATGAGCTTTCC	R: ACGGTGAACTTCTCGCACAT	281
CyclinD1	F: TGGGCTCCCTGAGTTGACTA	R: GCCGGGGAGGTTTCGATTTT	120
CyclinD2	F: ACAGTTTGCCAGAGCAGGTT	R: CATGCGCTGCACATAGTTCC	194
CyclinD3	F: CTGGTCTCGGTGATAGCG	R: GACGAAAGTGTAGTCTGTGGC	135
p21	F: GATCCCAGGTTGGGTGAGAAAT	R: GGCCTTCATGGTAAGTGGCA	274
p27	F: CTGGAAGGCAGGTACGAGTG	R: TGAGGAGAATCGTCGGT	281

### 2.10 Statistical analysis

All experimental data were organized and recorded using Excel 2023 (Microsoft, USA). Statistical analyses were performed using IBM SPSS Statistics software (v.27.0). One-way analysis of variance (ANOVA) was used to compare differences among groups, with Bonferroni method applied for post-hoc tests. Pearson correlation analysis was used to examine the correlations between variables. Data are presented as mean±standard deviation (S. D.), with *p* < 0.05 considered statistically significant. Graphs were generated using GraphPad Prism software (v.5). Protein–protein interactions among differentially expressed genes were analyzed using the STRING database and visualized using Cytoscape software. Correlation analysis and result visualization were conducted using R language (v.4.2.3).

## 3 Results

### 3.1 Effect of overfeeding with different types of sugar on liver index, serum indices and fatty acid of goose

**Figure 1:**
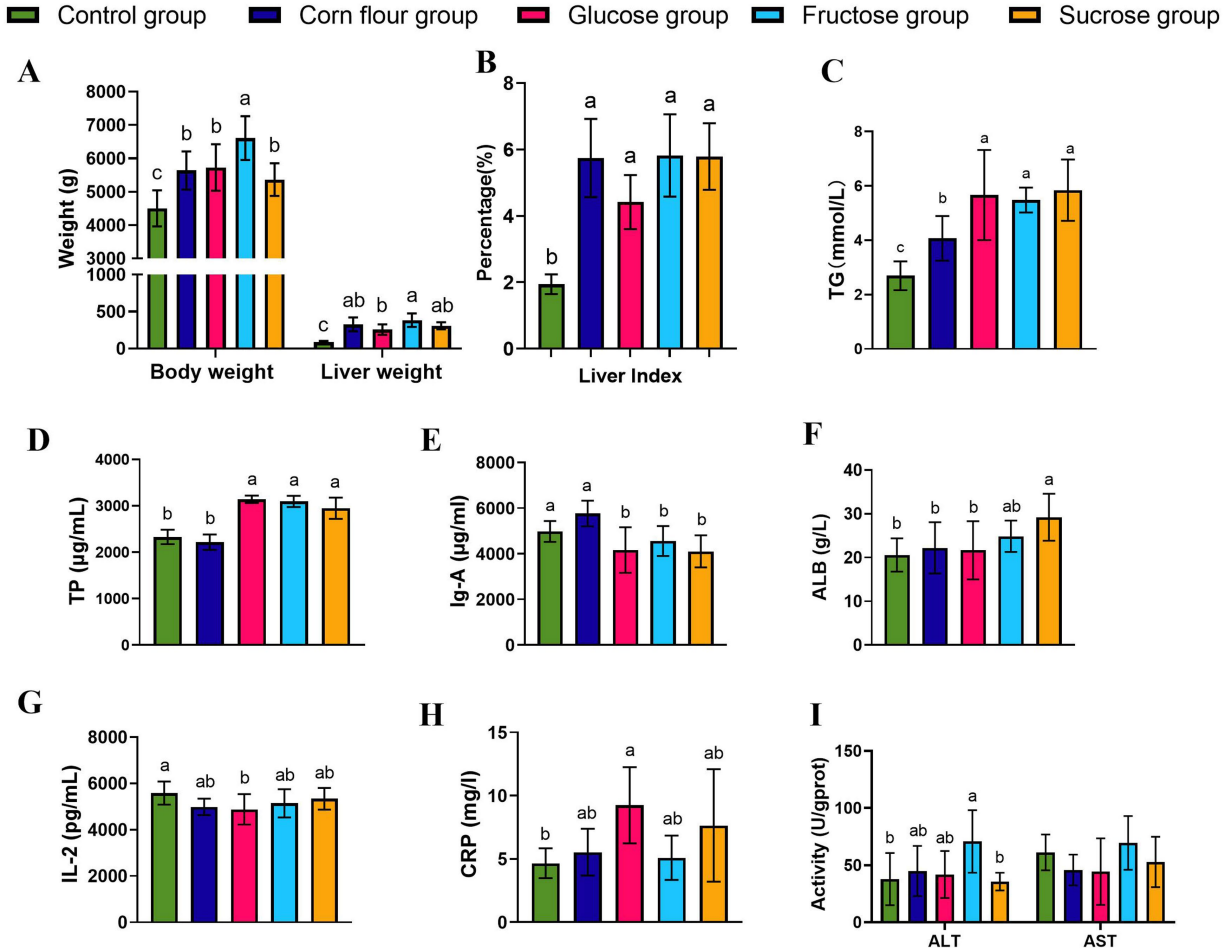
Effects of supplementing sugar on body weight, liver volume, liver index, and biochemical indicators related to goose liver function in feeding geese. **(A)** Body weight and liver weight (*n* = 6); **(B)** Liver index (*n* = 6); **(C)** TG(*n* = 6); **(D)**Total protein TP (*n* = 6); **(E)** Serum immunoglobulin A IgA (*n* = 6); **(F)** albumin ALB (*n* = 6); **(G)** Interleukin 2, IL-2(*n* = 6); **(H)** C - reactive protein CRP (*n* = 5); **(I)** Serum ALT and serum AST (*n* = 6). Different lowercase letters (a–c) above the bar indicate differences between treatments (*p* < 0.05). Values are mean ± standard deviation.

To visually observe the influence of overfeeding dietary supplementation with the three different types of sugar on the goose fatty liver formation, the body weight and liver weight of geese were measured. The geese of the fructose group had the heaviest body weight (Figure 1A). The liver weight of geese of fructose group was heavier than that of glucose group. The ratio of liver to body weight of the four overfeeding groups were higher than that of the control group (*p <* 0.05) (Figure 1B). The results suggested that fructose had better promotion for lipid deposition in the liver of overfed geese. Furthermore, the serum indices of the geese were detected. The TG level of the corn overfeeding group was lower than that of the glucose, fructose, and sucrose overfeeding groups (*p* < 0.05) (Figure 1C). The results of serum biochemical indices were presented in Figures 1D–I. Compared with geese of the corn flour and control groups, geese overfed with corn flour supplemented with sugar had higher content of TP. Compared with the control group, the glucose group was lower in serum IgA and IL-2 levels, in contrast, higher in CRP level. Additionally, the fructose group showed higher ALT activity than the control group (*p <* 0.05), but had no difference (*p >* 0.05) in AST activity. The obvious changes in the concentrations of IgA, AST, ALT and CRP of the four overfeeding groups suggested that diets supplemented with different types of sugar may have a potential effect on the inflammation in geese. Dietary sugar supplementation significantly altered the hepatic fatty acid profile in overfed geese (Table 2). All sugar-supplemented groups (glucose, fructose, sucrose) and the corn flour group exhibited 2.3- to 7.9-fold higher TFA compared to controls, confirming successful induction of hepatic steatosis. Fructose and sucrose groups showed the highest TFA accumulation though differences among sugar types were not statistically significant (*p* > 0.05). Compared with the control group, the SFA, UFA and MUFA of all sugar supplementation groups and corn flour groups were increased (*p* < 0.05). PUFA levels remained stable across groups, suggesting sugar types primarily modulate *de novo* lipogenesis rather than PUFA metabolism.

**Table 2:** Effect of different types of sugar (glucose, fructose, and sucrose) on fatty acid composition.

Items	Control group	Corn flour group	Glucose group	Fructose group	Sucrose group
TFA	3.86 ± 1.741^b^	24.096 ± 8.34^a^	26.122 ± 5.722^a^	30.558 ± 7.736^a^	30.047 ± 4.525^a^
SFA	1.364 ± 0.608^b^	6.987 ± 2.71^a^	7.307 ± 1.752^a^	8.835 ± 2.699^a^	8.657 ± 2.09^a^
UFA	2.496 ± 1.136^b^	17.109 ± 5.653^a^	18.816 ± 4.034^a^	21.724 ± 5.241^a^	21.39 ± 2.752^a^
MUFA	1.433 ± 1.02^b^	15.467 ± 5.441^a^	17.051 ± 3.82^a^	20.092 ± 5.282^a^	19.791 ± 2.912^a^
PUFA	1.063 ± 0.14^6^	1.643 ± 0.27	1.765 ± 0.255	1.631 ± 0.315	1.6 ± 0.272

Statistical results of fatty acid composition (unit: g/100 g for SFA, MUFA, PUFA, and TFA). Values represent means ± SD, *n* = 6. Different lowercase letters within the same row indicate significant differences among treatments at *p* < 0.05. Abbreviations: TFA, total fatty acids; SFA, saturated fatty acids; MUFA, monounsaturated fatty acids; PUFA, polyunsaturated fatty acids.

### 3.2 Response of DEGs associated with proliferation and inflammation to overfeeding dietary supplements with different types of sugar

**Figure 2:**
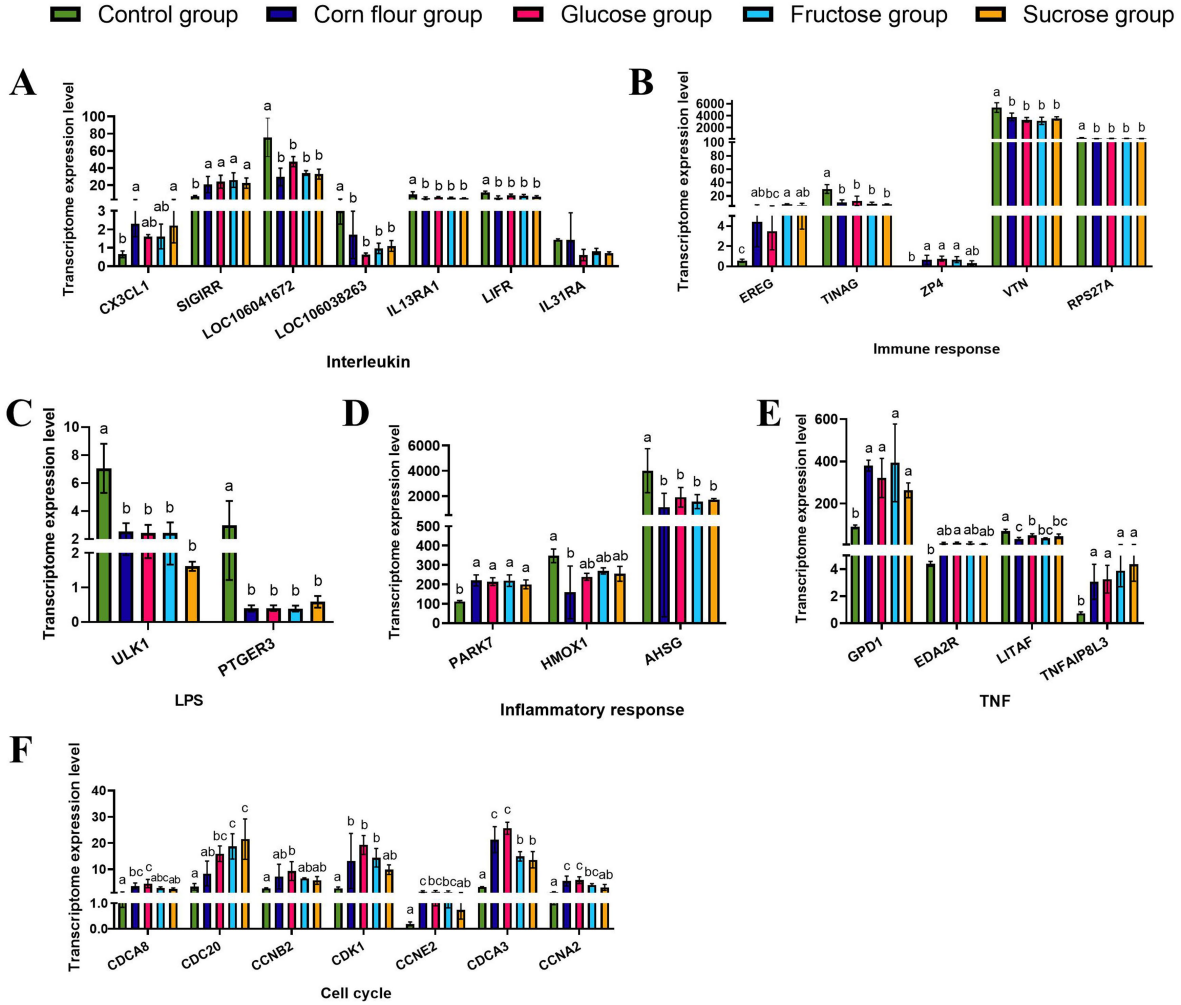
Diverse effects of glucose or fructose or sucrose on genes expression in categories and transcriptional regulators of interleukin, immune response, inflammatory response, LPS response, cell cycle and the different effects of TNF signalling pathways by RNA-sequencing. **(A)** *CX3CL1*, *SIGIRR*, *EREG*, *LOC106041672*, *LOC106038263*, *IL13RA1*, *LIFR*, and *IL31RA* are associated with cytokine production. **(B)** *EREG*, *TINAG*, *ZP4*, *VTN*, and *RPS27A* are associated with immune response. **(C)** *ULK1* and *PTGER3* are associated with lipopolysaccharide (LPS). **(D)** *PARK7*, *HMOX1*, and *AHSG* are associated with inflammatory response. **(E)** *GPD1*, *EDA2R*, *LITAF*, and *TNFAIP8L3* are associated with TNF. **(F)** *CDCA8*, *CDC20*, *CCNB2*, *CDK1*, *CCNE2*, *CDCA3*, and *CCNA2* are associated with the cell cycle. Values are expressed as the mean ± standard deviation (*n* = 3). Different lowercase letters (a–c) on the charts indicate differences between treatment groups (*p <* 0.05).

The specific data of DEGs involved in cell cycle and inflammation were presented in Supplementary Table S1. In the glucose group, the interleukin-associated genes (*LOC106041672*, *LOC106038263*, *IL13RA1*, and *LIFR*) were down-regulated, except for *SIGIRR*. Genes involved in immune response (*TINAG*, *VTN*, and *RPS27A*) were also down-regulated. Furthermore, genes associated with LPS response (*ULK1* and *PTGER3*) were down-regulated. As to inflammation response genes, *PARK7* connected with oxidative stress and apoptosis was up - regulated, while *AHSG* was down-regulated. Genes related to TNF signaling pathways (*GPD1* and *TNFAIP8L3*) were up-regulated. In the fructose group, sucrose group and corn flour group, the regulation trend of the above mentioned DEGs expression levels was consistent with the glucose group. Additionally, genes involved in the cell cycle, such as *CDCA8*, *CDC20*, *CCNB2*, *CDK1*, *CCNE2*, *CDCA3*, and *CCNA2*, were all up-regulated in the glucose group. The fructose group and sucrose group both up-regulated the expression of *CDC20* (Figure 2). These findings indicated that dietary supplementation with glucose may promote cell proliferation by enhancing the expression of these cell cycle regulators.

### 3.3 Correlation analysis of DEGs involved in lipid metabolism, proliferation, and inflammation between control and treatment groups

**Figure 3:**
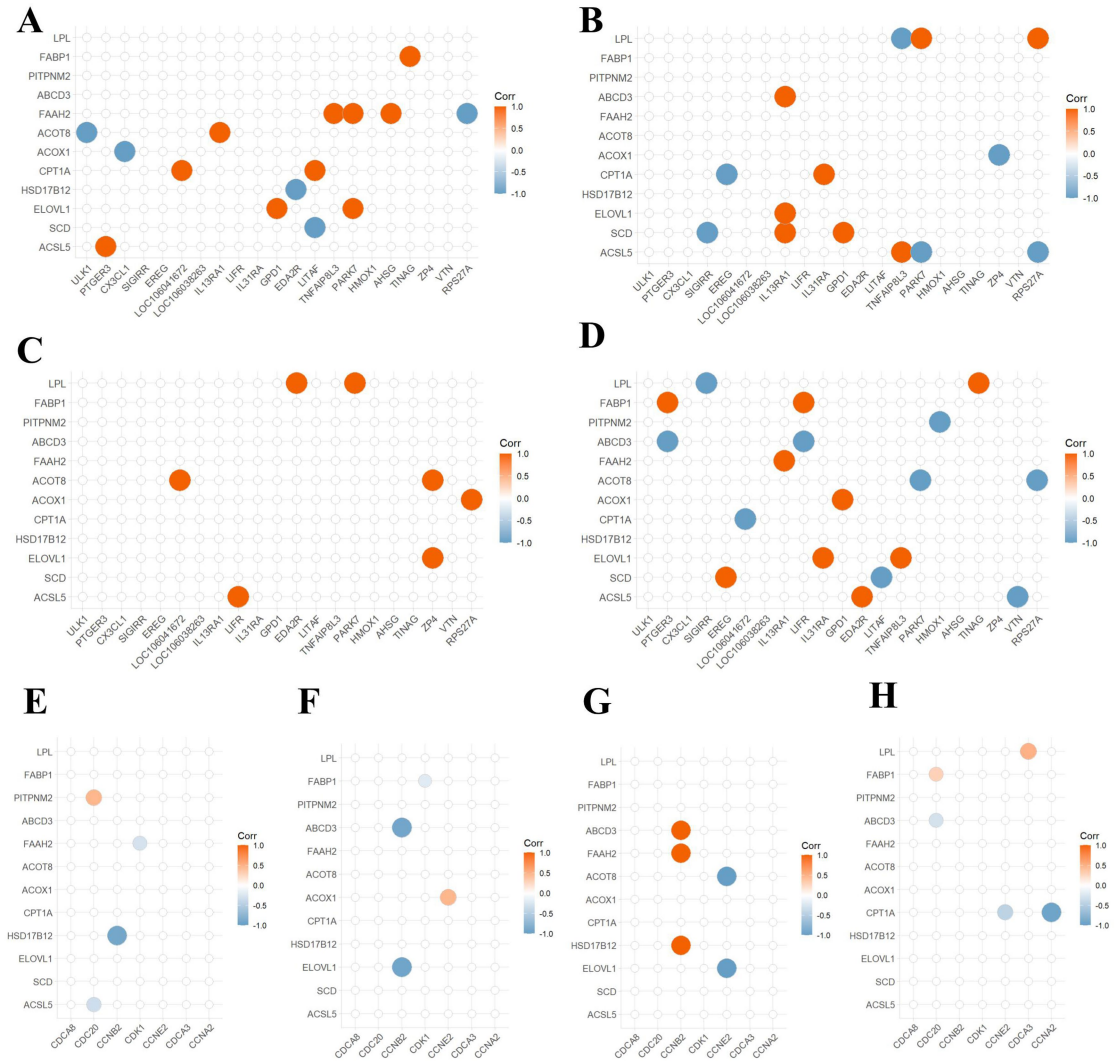
Correlation analysis between lipid metabolism genes and DEGs in inflammation, immunity, and cell cycle. **(A,E)** Correlation analysis for the cornmeal group. **(B,F)** Correlation analysis for the glucose group. **(C,G)** Correlation analysis for the fructose group. **(D,H)** Correlation analysis for the sucrose group. The horizontal axis is interpreted as in Figure 5. Genes on the vertical axis such as *CPT1A*, *ACOX1*, *ACOT8*, and *FAAH2* are related to lipid oxidation. Genes *ABCD3* and *PITPNM2* are related to lipid transport. Genes *FABP1* and *LPL* are related to lipid catabolism. The size of the circles and the darkness of the color indicate the strength of the correlation. The larger the circle and the darker the color, the stronger the correlation. Red indicates positive correlation, and blue indicates negative correlation.

Based on transcriptomic sequencing analysis, DEGs involved in lipid metabolism (Supplementary Table S2) and those involved in inflammatory immune response and cell cycle were screened out. Therefore, a detailed correlation analysis was conducted. In the corn flour group as illustrated (Figures 3A,E), fatty acid amide hydrolase (*FAAH2*) was positively correlated with *LITAF*, *TNFAIP8L3* and *AHSG*, and negatively correlated with *RPS27A*. Acyl coenzyme A thioesterase (*ACOT8*) was positively correlated with *ULK1*. *CPT1A* had a positive correlation with *LOC106041672* and *LITAF*. Elongation of very long chain fatty acids protein (*ELOVL1*) was positively correlated with *GPD1* and *PARK7*. 17beta-estradiol 17-dehydrogenase (*HSD17B12*) was positively correlated with *CCNB2*. In Figures 3B,F, correlation analysis of the glucose group showed that lipoprotein lipase (*LPL*) was positively correlated with *PARK7*and *RPS27A*, and was negatively associated with *TNFAIP8L3*. *ASCL5* and *LPL* had opposite correlations with these three genes. ATP-binding cassette sub-family D member (*ABDC3*) had a positive correlation with *IL13RA1*, and negative correlation with *CCNB2*. *CPT1A* was negatively correlated with *EREG* and positively correlated with *IL31RA*. In the fructose group, there was a positive correlation between lipid metabolism-related DEGs and inflammatory response-related DEGs. *CCNB2* was positively correlated with *ABCD3*, *FAAH2* and *HSD17B12*. *CCNE2* had a negative correlation with *ACOT8* and *ELOVL1* (Figures 3C,G). The results of Figures 3D,H indicated that in the sucrose group, fatty acid - binding protein (*FABP1*) was positively correlated with *PTGER3* and *LIFR*, and *ABCD3* and *FABP1* had opposite correlations with these genes. *ACOT8* was negatively correlated with *PARK7*, *RPS27A*, and *CPT1A* had a negative correlation with *LOC106041672*, *CCNE2* and *CCNA2*. *ELOVL1* was positively correlated with *IL13RA1*and *TNFAIP8L3*. In summary, *CPT1A* showed varying degrees of correlation with DEGs involved in immune and inflammatory responses and cell cycle regulation.

### 3.4 Glucose and fructose alter proliferation and inflammatory factor expression in goose hepatocytes

**Figure 4:**
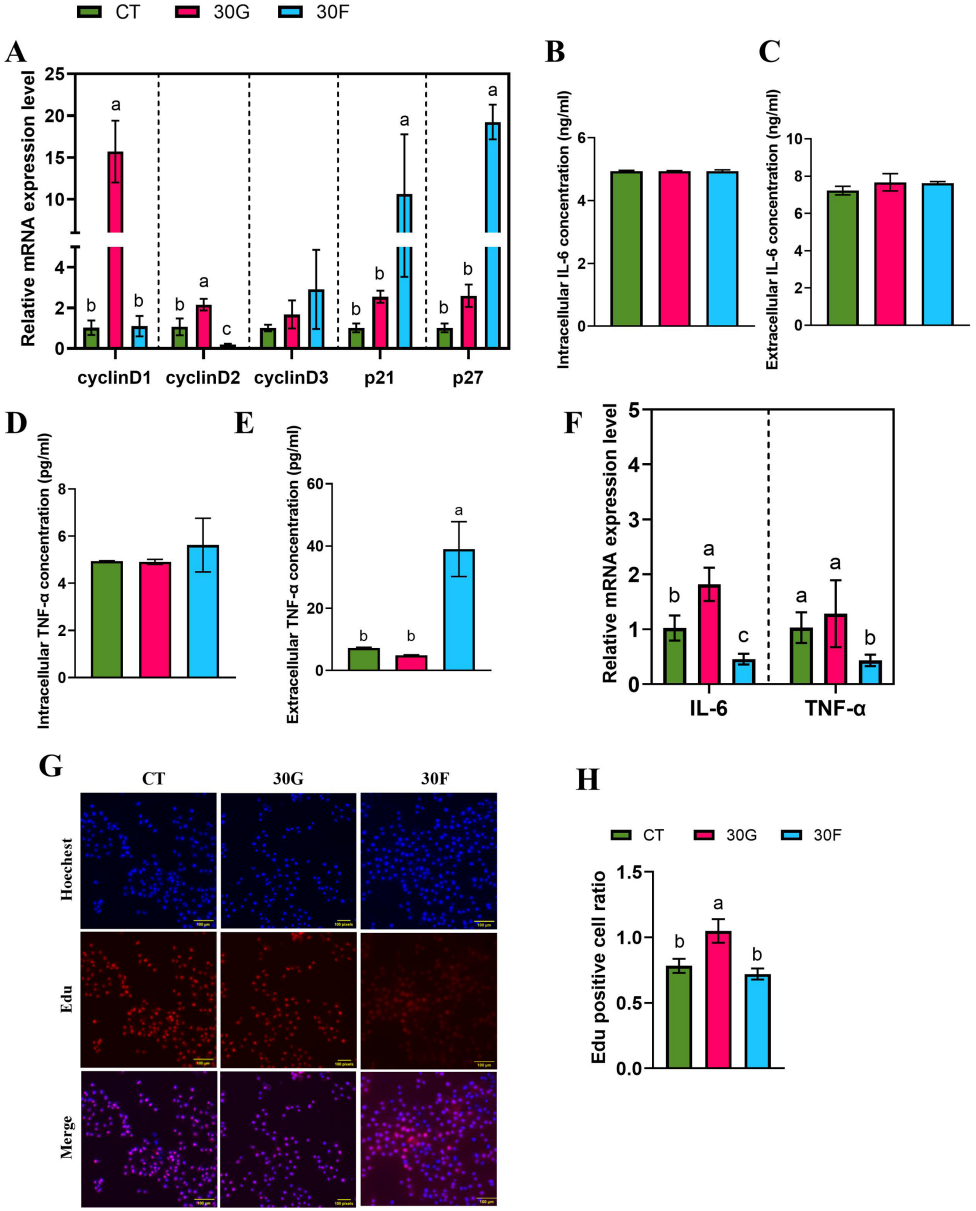
Effects of glucose and fructose on the mRNA expression of cell proliferation related genes and proinflammatory cytokines in goose liver. **(A)** mRNA expression levels of *CyclinD1*, *CyclinD2*, *CyclinD3*, *p21* and *p27*, normalized as GAPDH. **(B)**. Intracellular IL-6 concentration (ng/ml). **(C)**. Extracellular IL-6 concentration (ng/ml). **(D)**. Intracellular TNF-α concentration (pg/mL). **(E)**. Extracellular TNF-α concentration (pg/mL). **(F)** The relative expression of *TNF-α* and *IL-6*. **(G, H)**. EdU kits were used to detect EdU staining-positive hepatocytes. Abbreviations: CT = Control group; 30G = glucose (30 mmol/L); 30F = fructose (30 mmol/L). EdU (red), DAPI (blue); *n* = 3. Different lowercase letters (a–c) above the bar indicate differences between treatments (*p <* 0.05).

To further investigated the effects of glucose and fructose on the proliferation of goose primary hepatocytes. The results showed that 30 mmol/L glucose significantly stimulated the mRNA expression of proliferation-related genes CyclinD1 and CyclinD2 (*p* < 0.05). In the 30 mmol/L fructose group, the mRNA expression of CyclinD2 was significantly reduced, while the expression of p21 and p27 was markedly increased (*p < 0.05*; Figure 4A). Additionally, EdU assay results demonstrated that the 30 mmol/L glucose group exhibited an increased cell proliferation rate (*p < 0.05*; Figures 4G,H). By measuring the intracellular and extracellular concentrations of *TNF-α* and *IL-6*, it was found that the 30 mmol/L fructose group increased the extracellular concentration of *TNF-α* (Figures 4B–E). Notably, the 30 mmol/L fructose group inhibited the mRNA expression of *TNF-α* and *IL-6*, whereas 30 mmol/L glucose treatment could stimulate *IL-6* mRNA expression (Figure 4F).

### 3.5 CPT1A interference combined with glucose or fructose modulates hepatocyte proliferation and inflammatory factors

**Figure 5:**
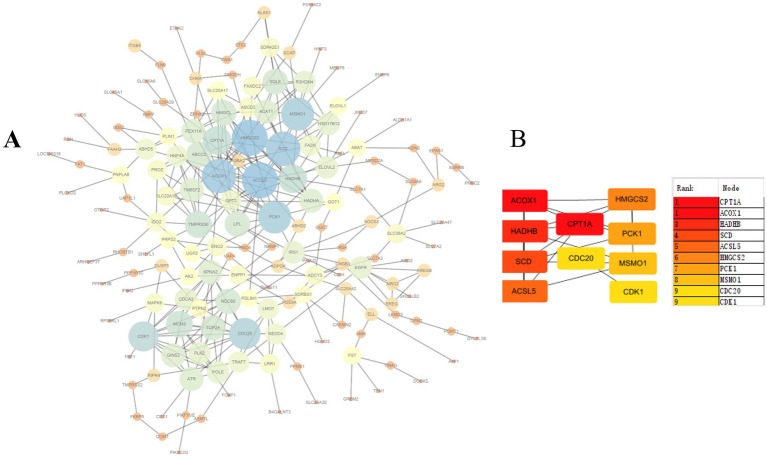
Protein–protein interaction (PPI) network. **(A)** The network consists of 161 nodes and 323 edges. **(B)** Ranking of top 10 hub genes in the PPI network using MCC (Maximal clique centrality) method.

The STRING database was utilized to construct a protein–protein interaction (PPI) network based on differentially expressed genes (DEGs) commonly identified across the four overfeeding groups. After removing genes without interaction relationships, the final PPI network comprised 161 nodes and 323 edges, indicating close functional associations among these genes (Figure 5A). The top 10 hub genes were re-ranked using the maximal clique centrality (MCC) method (Figure 5B). The MCC algorithm, based on maximal cliques, provides a comprehensive assessment of node centrality by considering not only connectivity but also topological positioning within the network. Analysis revealed that CPT1A exhibited high centrality. As the rate-limiting enzyme of fatty acid β-oxidation, CPT1A plays a crucial role in hepatic lipid metabolism regulation, and its expression changes may directly influence hepatic fat deposition in geese.

**Figure 6:**
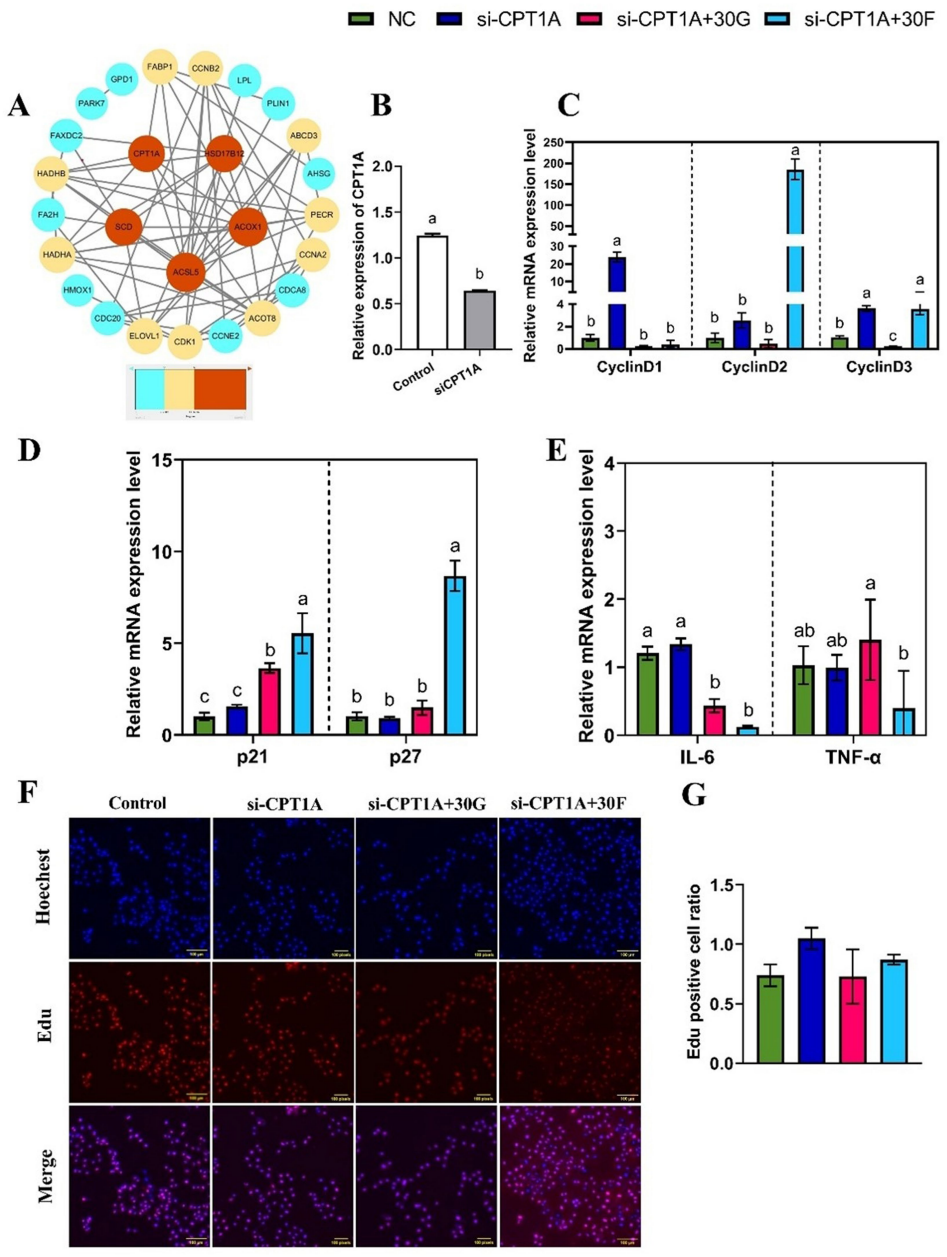
Effects of glucose and fructose treatments and interference with *CPT1A* on inflammatory cytokines and cyclin in goose primary hepatocytes. **(A)** PPI network of DEGs, those genes are shown, gradient colors indicate the strength of the DEGs correlation. **(B)** mRNA expression levels of *CPT1A* interference (si-*CPT1A*) and siRNA negative control (NC) in primary goose hepatocytes. **(C, D, E)** mRNA expression levels of *CyclinD1*, *CyclinD2*, *CyclinD3*, *p21*, *p27*, *TNF-α*, and *IL-6*. **(F, G)** EdU staining-positive hepatocytes detected by the EdU assay kit. Abbreviations: Control, control group; si-*CPT1A*, negative control; si-*CPT1A* + 30G = CPT1A interference combined with 30 mmol/L glucose treatment; si-*CPT1A* + 30F=*CPT1A* interference combined with 30 mmol/L fructose treatment. EdU (red), Hoechst (blue); *n* = 3. Different lowercase letters on top of the bar charts indicate differences between treatments (*p <* 0.05).

To further explore potential regulatory relationships between lipid metabolism and genes associated with cell proliferation and inflammation, a PPI network of differentially expressed genes was constructed using the STRING database (Figure 6A) and visualized using Cytoscape software. Notably, key genes such as *ACSL5*, *ACOX1*, *SCD*, *HSD17B12*, and *CPT1A* were closely associated with lipid metabolism pathways. Based on preliminary research data, *CPT1A* as hypothesized to play a significant role in regulating lipid metabolism in goose hepatocytes. Therefore, *CPT1A* was selected for subsequent cellular experiments.

In this experiment, a specific siRNA vector plasmid targeting CPT1A (si-CPT1A) was transfected to knock down CPT1A expression, with a negative control plasmid-transfected group (NC) serving as the control. RT-qPCR results confirmed successful interference of CPT1A gene expression in goose primary hepatocytes (Figure 6B). The si*-CPT1A*-treated hepatocytes group up-regulated the mRNA expression levels of *CyclinD1* and *CyclinD3* (*p < 0.05*), while there was no difference (*p > 0.05*) in the expression levels of *p21* and *p27* compared with the control group. Under the co-treatment with glucose and si-*CPT1A*, the mRNA expression level of *CyclinD3* reduced, while the mRNA expression level of *p21* increased (*p <* 0.05). Furthermore, co-treatment with fructose and si-*CPT1A* led to higher expression levels of *CyclinD2*, *CyclinD3*, *p21*, and *p27* compared with the treatment of si*-CPT1A* group (*p <* 0.05; Figures 6C,D). The EdU assay showed that co - treatment with 30 mmol/L glucose or fructose and si-*CPT1A* had no difference (*p >* 0.05) on cell proliferation rates (Figures 6F,G). Further investigation was conducted to determine whether si-*CPT1A* could affect the inflammatory response of hepatocytes induced by different sugar. The results indicated that co-treatment with glucose or fructose and si-*CTP1A* decreased the expression of *IL-6* (*p <* 0.05; Figure 6E). It was demonstrated that si*-CPT1A* could promote goose hepatocyte proliferation. In addition, co-treatment with sugar and si*-CPT1A* could decreased the expression of the inflammatory factor *IL-6*, but had no effect on goose hepatocyte proliferation.

## 4 Discussion

To further investigate the effects of sugar on hepatocytes proliferation and inflammatory response during goose fatty liver formation, a 3-week overfeeding experiment was carried out and hepatic steatosis in geese were successfully induced. The results showed that the body weight, liver weight, and liver-to-body ratio of all overfed geese increased after overfeeding, which was consistent with the previous findings reported by Liu, et al. (23). Comparative studies across livestock species have revealed species-specific differences in the effects of dietary sugar supplementation on body weight. The inclusion of fructose in finishing pig diets showed no significant impact on growth performance or inflammatory responses (24), although other studies have demonstrated that high-fructose feeding upregulates hepatic *de novo* lipogenesis-related enzymes (acetyl-CoA carboxylase and fatty acid synthase) expression in pigs, with adipose tissue serving as the primary site for lipogenesis in this species (25). Notably, in our study, the fructose-fed group exhibited the most pronounced effects, displaying the highest body weight and liver weight, which may be attributed to the force-feeding protocol delivering fructose intake levels far exceeding natural consumption in geese. The hepatic metabolism of fructose through fructokinase bypasses the rate-limiting phosphofructokinase step, resulting in more efficient lipogenesis. Wei et al. (18) observed marked hepatomegaly in overfed geese, with histopathological analysis (H&E staining) revealing severe microvesicular steatosis characterized by uniformly enlarged hepatocytes and displaced nuclei - particularly in fructose- and sucrose-supplemented groups. These morphological changes directly correlate with our biochemical measurements showing 2.3- to 7.9-fold higher hepatic triglyceride (TG) accumulation in sugar-fed groups (Table 2). Transaminases (ALT and AST) and CRP serve as the biomarkers of liver damage and inflammatory responses, which provided important insights into liver panorama (26, 27). Overfeeding dietary supplement with 10% glucose decreased the levels of IgA and IL*-*2, and elevated the CRP level in serum. Additionally, overfeeding dietary supplement with 10% fructose increased ALT level. These results suggested that different sugars may have distinct impacts on goose liver health and inflammation. Glucose might weaken the immune system of overfed geese, while fructose might cause liver damage. In terms of inflammation and immunity, the expression of inflammatory genes was quantified. Notably, in the four overfeeding groups, inflammatory genes such as *ULK1*, *PTGER3*, and *AHSG* showed a downward trend during goose fatty liver formation. ULK1, as an autophagy - related gene, may promote autophagy and reduce inflammation when its activity increases (28). The activation of PTGER3 can inhibit the production of inflammatory mediators and exert anti - inflammatory effects (29). AHSG suppresses inflammation by activating macrophages (30). In addition, the expression levels of *PARK7* and *TNFAIP8L3* were up - regulated, which played a crucial role in regulating the inflammatory response (31, 32). In the correlation analysis between DEGs related to lipid metabolism and inflammation, *LPL* was positively correlated with *PARK7* and negatively correlated with *TNFAIP8L3* in the glucose group. Combined with the above findings, it was indicated that glucose may modulate the interaction between lipid metabolism and inflammatory responses through specific gene regulation.

In addition, *LPL* was also positively correlated with *PARK7* in fructose group, indicating that fructose may promote lipid accumulation and inflammation through the up-regulation of specific genes. In the digestion system, sucrose is broken down into glucose and fructose for utilization. Subsequently, goose primary hepatocytes were treated with glucose or fructose *in vitro*. The results manifested that fructose treatment downregulated the expression levels of *TNF-α* and *IL-6*. In rodent studies, both glucose and fructose, particularly when administered in high-fat diets, are well-established inducers of hepatic steatosis, insulin resistance, and pro-inflammatory responses (15, 33). For instance, Park et al. (12) demonstrated that dietary and genetic obesity in mice promote liver inflammation and tumorigenesis by enhancing IL-6 and TNF expression. This typical mammalian pro-inflammatory outcome starkly contrasts with our observation in geese. We observed attenuated inflammatory cytokine expression in fructose-treated goose hepatocytes. This key discrepancy may be attributed to fundamental differences in waterfowl physiology. Geese, as opposed to rodents, are evolutionarily adapted to develop significant hepatic steatosis as a natural energy reserve for migration. This adaptation likely includes suppressed inflammatory pathways to tolerate massive lipid accumulation without incurring severe damage, a mechanism not prevalent in mammals (7, 10). As evidenced by in vitro findings that goose hepatocytes also exhibit a tolerance mechanism when incubated with high glucose, resisting high glucose-induced inflammation (20). In particular, the addition of fructose may involve the enhancement of anti-inflammatory pathways or the differential activation of immune cells in the avian hepatic microenvironment.

Glucose and fructose not only provide ATP energy, but also serve as an important carbon source for the synthesis of lipids and non - essential amino acids in cell, thus, supporting cell growth (34). Cell cycle regulatory proteins play a crucial role in cell proliferation regulation, which directly influence cell growth. The cyclin D family of cell cycle proteins positively regulates cell proliferation (35). *P21* and *p27* are also important cell cycle regulatory factors that negatively regulate the cell cycle (36). In goose primary hepatocyte culture experiment, glucose increased the mRNA expression of proliferation - related genes *CyclinD1* and *CyclinD2*, further supporting the positive role of glucose in promoting cell proliferation, which was consistent with previous study reported by Wei et al. (37). In contrast, fructose upregulated the expression of *p21* and *p27*, which suggested that fructose may inhibit the proliferation of goose hepatocytes. Thus, it was concluded that glucose and fructose might have opposite effects on the expression of cell proliferation. This observation was consistent with previous study involved in hepatocellular carcinoma (HCC) cells, which reported by Dewdney et al. (38). The reason may be that fructose metabolism consumes ATP and leads to the accumulation of uric acid (38). Additionally, overfeeding dietary supplement with 10% glucose upregulated the expression of cell cycle genes, such as *CDCA8*, *CDC20*, *CCNB2*, *CDK1*, *CCNE2*, *CDCA3*, and *CCNA2*. These observations collectively indicated that glucose directly promotes the proliferation of goose hepatocytes.

Carnitine palmitoyl transferase I (CPT1), located on the inner mitochondrial membrane, reversibly catalyzes the formation of acylcarnitine esters from specific chain-length acyl-CoAs and carnitine (39). As the most widely distributed isoform in the CPT1 family, the hepatic subtype CPT1A plays a decisive role in fatty acid β-oxidation (40). CPT1A is closely associated with fat deposition through its regulation of lipid metabolism and cell proliferation, although the underlying mechanisms remain elusive (41). Intriguingly, CPT1A exhibits bidirectional regulatory effects on cell proliferation across different disease models. In nasopharyngeal carcinoma cells, shRNA-mediated knockdown of CPT1A significantly inhibits proliferative capacity, which is closely linked to energy deficiency caused by impaired fatty acid oxidation (42). Similarly, CPT1A knockdown downregulates proliferation-related genes in goat intramuscular preadipocytes, and CPT1A is regulated by the MAPK signaling pathway to influence their proliferation (41). Conversely, in clear cell renal carcinoma models, CPT1A inhibition enhances proliferation while its overexpression suppresses proliferation (43). Correspondingly, Li et al. (44) observed increased proliferation in chicken intramuscular preadipocytes following CPT1A knockdown, whereas overexpression reduced proliferation, suggesting that CPT1A’s effects depend on cell-specific metabolic characteristics and microenvironment. The cell-specificity of CPT1A function is further underscored by the divergent responses between avian and mammalian models. In goats and other ruminants, lipid metabolism is heavily influenced by volatile fatty acids derived from rumen fermentation, leading to a different metabolic setpoint in their tissues compared to monogastric species like geese and chickens. More importantly, the goose hepatocyte, as the primary site of *de novo* lipogenesis in waterfowl, possesses a unique metabolic identity that prioritizes lipid storage over oxidation during the overfeeding period. This anabolic priority may explain why CPT1A knockdown, which impairs fatty acid oxidation, paradoxically signals a pro-proliferative state in goose hepatocytes, a response not commonly observed in mammalian hepatocytes that are not evolutionarily programmed for such extreme lipid storage. In our goose primary hepatocyte model, siRNA-mediated CPT1A knockdown upregulated CyclinD1 and CyclinD3 mRNA levels. However, co-treatment with glucose and si-CPT1A reversed this pro-proliferative effect, evidenced by downregulated CyclinD1/D3 and upregulated cell cycle inhibitor p21 mRNA levels, indicating that CPT1A function may be modulated by carbohydrate metabolism. This phenomenon likely stems from CPT1A knockdown-induced impairment of fatty acid oxidation, resulting in energy metabolic imbalance and cell cycle arrest triggered by accumulated carbohydrate metabolites. CPT1A expression is closely associated with inflammatory status, as patients with CPT1A deficiency often exhibit hepatic steatosis and enhanced systemic inflammation (45). Our results demonstrate that glucose treatment significantly elevates IL-6 mRNA expression in goose primary hepatocytes, an effect attenuated by CPT1A knockdown, suggesting glucose may partially activate inflammatory signaling through CPT1A-independent pathways. Conversely, combined fructose and si-CPT1A treatment markedly reduced TNF-*α* and IL-6 mRNA levels, indicating CPT1A differentially mediates glucose- and fructose-induced inflammatory responses through distinct mechanisms.

Collectively, our findings, when contrasted with studies in mammalian models, emphasize the unique hepatic adaptive mechanisms in waterfowl. The goose’s capacity to orchestrate massive lipid deposition while concurrently suppressing overt inflammatory responses and modulating cell cycle checkpoints represents a distinct physiological strategy. This strategy diverges significantly from the pathological pathways observed in mammalian NAFLD, where inflammation and fibrosis are hallmarks. Therefore, the goose not only serves as a valuable model for understanding extreme hepatic steatosis but also provides evolutionary insights into how livers can tolerate lipotoxicity through species-specific adaptations.

## 5 Conclusion

In summary, overfeeding with different sugars significantly promoted fatty liver formation in geese, with fructose showing the most potent effect on lipid deposition. Serum biochemical analysis indicated that overfeeding disrupted systemic homeostasis, with glucose impairing immune parameters and fructose increasing liver injury markers (ALT). *In vitro*, fructose treatment attenuated the expression of key pro-inflammatory cytokines (TNF-*α* and IL-6) in hepatocytes, which might contribute to the relatively low-grade inflammation in goose fatty liver despite severe steatosis. However, it is crucial to emphasize that this observed anti-inflammatory effect is localized and does not negate the overall pathophysiological stress and health burdens imposed by the force-feeding regimen itself on the whole animal. Furthermore, the CPT1A gene was identified as playing a key mediating role in the regulation of hepatocyte proliferation and inflammatory cytokine expression by dietary sugars.

## Data Availability

The data presented in this study have been deposited and are publicly available via the following DOI: https://doi.org/10.6084/m9.figshare.30579401.
