# Supplementary material for: Study on the effect of different types of sugar on proliferation and inflammatory in goose fatty liver
Source: Front Vet Sci. 2025 Nov 25;12:1625050. doi: 10.3389/fvets.2025.1625050 (PMC12685652; doi:10.3389/fvets.2025.1625050)
Supplement: Supplementary file 1 [file Table_1.DOCX]

**Table S1** **Expression of DEGs involved in proliferation and inflammation from transcriptome analysis**

| Items | Control group | Corn flour group | Glucose group | Fructose group | Sucrose group |  |
| --- | --- | --- | --- | --- | --- | --- |
| ULK1 | 7.05±1.75 ^a^ | 2.54±0.6 ^b^ | 2.44±0.58 ^b^ | 2.43±0.77 ^b^ | 1.62±0.13 ^b^ |  |
| PTGER3 | 2.97±1.75 ^a^ | 0.39±0.09 ^b^ | 0.4±0.08 ^b^ | 0.39±0.09 ^b^ | 0.59±0.17 ^b^ |  |
| CX3CL1 | 0.65±0.18 ^b^ | 2.3±0.7 ^a^ | 1.62±0.1 ^ab^ | 1.62±0.67 ^a^ | 2.21±0.94 ^ab^ |  |
| SIGIRR | 6.75±1.05 ^b^ | 20.57±9.53 ^a^ | 23.93±7.57 ^a^ | 25.84±8.49 ^a^ | 22.3±5.98 ^a^ |  |
| LOC106041672 | 75.68±22.49 ^a^ | 29.45±10.23 ^b^ | 47.38±5.92 ^b^ | 34.12±2.73 ^b^ | 32.79±5.84 ^b^ |  |
| LOC106038263 | 2.99±0.72 ^a^ | 1.7±1.28 ^b^ | 0.63±0.09 ^b^ | 0.97±0.28 ^b^ | 1.1±0.29 ^b^ |  |
| EREG | 0.55±0.16 ^c^ | 4.41±2.46 ^ab^ | 3.47±1.83 ^bc^ | 7.43±1.03 ^ab^ | 6.34±2.65 ^a^ |  |
| IL13RA1 | 9.23±2.98 ^a^ | 4.64±1.45 ^b^ | 6.01±0.62 ^b^ | 5.16±0.71 ^b^ | 4.69±0.36 ^b^ |  |
| LIFR | 11.43±1.56 ^a^ | 5.38±2.02 ^b^ | 7.86±1.6 ^b^ | 7.61±1.43 ^b^ | 6.26±1.35 ^b^ |  |
| IL31RA | 1.44±0.05 | 1.43±1.47 | 0.61±0.3 | 0.81±0.16 | 0.72±0.07 |  |
| GPD1 | 89.56±8.52 ^b^ | 379.57±26.06 ^a^ | 321.14±92.98 ^a^ | 392.8±184.29 ^a^ | 262.65±35.11 ^a^ |  |
| EDA2R | 4.39±0.18 ^b^ | 10.74±4.81 ^ab^ | 12.86±4.16 ^a^ | 11.91±6.28 ^ab^ | 7.93±2.38 ^ab^ |  |
| LITAF | 69.2±8.77 ^a^ | 31.49±7.07 ^c^ | 48.73±8.17 ^b^ | 35.46±1.37 ^bc^ | 45.03±10.69 ^bc^ |  |
| TNFAIP8L3 | 0.74±0.1 ^b^ | 3.06±1.3 ^a^ | 3.25±1.03 ^a^ | 3.88±1.18 ^a^ | 4.36±1.25 ^a^ |  |
| PARK7 | 112.04±4.5 ^b^ | 220.58±28.5 ^a^ | 214.13±19.99 ^a^ | 219.1±29.71 ^a^ | 200.1±23.02 ^a^ |  |
| Items | | Control group | Corn flour group | Glucose group | Fructose group | Sucrose group |
| HMOX1 | 347.63±35.25^a^ | 158.99±135.79 ^b^ | 239.56±17.94 ^ab^ | 269.36±15.87 ^ab^ | 254.37±38.39 ^ab^ |  |
| AHSG | 4010.67±1745.98^a^ | 1125.12±1092.54^b^ | 1907.59±777.12^b^ | 1567.11±554.63 ^b^ | 1702.03±102.75 ^b^ |  |
| TINAG | 30.42±6.48 ^a^ | 10.74±3.38 ^b^ | 12.74±7 ^b^ | 8.44±2.28 ^b^ | 7.51±0.68 ^b^ |  |
| ZP4 | 0.01±0.01 ^b^ | 0.65±0.45 ^a^ | 0.76±0.25 ^a^ | 0.67±0.31 ^a^ | 0.35±0.21 ^ab^ |  |
| VTN | 5371.25±799.87 ^a^ | 3798.62±655.68 ^b^ | 3324.84±389.79^b^ | 3150.05±617.7 ^b^ | 3566.58±279.15 ^b^ |  |
| RPS27A | 283.5±54.24 ^a^ | 165.96±16.35 ^b^ | 190.81±28.88 ^b^ | 202.16±11.02 ^b^ | 171.3±25.43 ^b^ |  |
| CDCA8 | 1.07±0.24 ^a^ | 3.6±1.14 ^ab^ | 4.51±1.58 ^c^ | 2.77±0.49 ^bc^ | 2.36±0.52 ^abc^ |  |
| CDC20 | 3.28±1.25 ^a^ | 8.35±4.74 ^c^ | 15.89±2.98 ^bc^ | 18.69±4.82 ^ab^ | 21.45±7.71 ^c^ |  |
| CCNB2 | 2.64±0.27 ^a^ | 7.17±4.7 ^ab^ | 9.3±3.58 ^b^ | 6.51±0.21 ^ab^ | 5.72±1.47 ^ab^ |  |
| CDK1 | 2.75±0.47 ^a^ | 13.14±10.52 ^ab^ | 19.25±3.61 ^b^ | 14.36±3.55 ^b^ | 9.83±1.84 ^b^ |  |
| CCNE2 | 0.19±0.07 ^a^ | 1.46±0.31 ^ab^ | 1.38±0.48 ^bc^ | 1.27±0.45 ^c^ | 0.73±0.36 ^bc^ |  |
| CDCA3 | 3.18±0.18 ^a^ | 21.22±5 ^b^ | 25.61±2.3 ^c^ | 14.91±1.79 ^c^ | 13.60±3.12 ^b^ |  |
| CCNA2 | 1.17±0.23 ^a^ | 5.49±1.86 ^ab^ | 5.83±1.29 ^c^ | 3.99±0.47 ^c^ | 3.03±1.14 ^bc^ |  |

Different lowercase letters (a-c) above the bar indicate differences between treatments (*p<0.05*). All results are presented as the mean ± standard deviation (S.D.) (n = 3).

**Table S2** **Expression of DEGs involved in lipid metabolism from transcriptome analysis**

| Items | Control group | Corn flour group | Glucose group | Fructose group | Sucrose group |
| --- | --- | --- | --- | --- | --- |
| ACSL5 | 17.75±3.48^b^ | 120.48±115.06^ab^ | 127.74±78.74^ab^ | 176.61±46.25^a^ | 124.48±54.89^ab^ |
| SCD | 292.43±66.36^b^ | 1049.96±527.99^ab^ | 1351.55±934^a^ | 1147.61±119.69^ab^ | 1760.03±436.2^a^ |
| ELOVL1 | 29.04±1.38^c^ | 52.7±50.65^bc^ | 142.99±68.22^a^ | 97.74±17.43^abc^ | 122.85±32.97^ab^ |
| HSD17B12 | 89.07±15.13^b^ | 357.56±71.82^a^ | 322.73±70.61^a^ | 284.78±16.55^a^ | 344.32±46.79^a^ |
| CPT1A | 19.35±5.68^c^ | 69.1±12.02^a^ | 57.08±18.2^ab^ | 50.81±5.64^ab^ | 48.58±7.79^b^ |
| ACOX1 | 54.75±3.97^c^ | 94.21±79.06^bc^ | 153.73±6.4^ab^ | 180.22±43.39^a^ | 134.38±9.59^ab^ |
| ACOT8 | 92.58±14.52^b^ | 217.29±17.56^a^ | 196.17±36.35^a^ | 225.87±19.07^a^ | 187.35±27.09^a^ |
| FAAH2 | 2.45±0.46^b^ | 7.26±2.64^b^ | 5.8±1.76^b^ | 5.27±1.05^ab^ | 6.89±0.57^b^ |
| ABCD3 | 7.24±1.55^b^ | 23.04±8.39^a^ | 17.94±4.39^a^ | 21.91±5.82^a^ | 18.44±2.22^a^ |
| PITPNM2 | 2.29±0.29 | 2.2±0.9 | 1.81±0.37 | 1.81±0.23 | 1.66±0.3 |
| FABP1 | 0^b^ | 2.34±0.68^b^ | 11.16±8.94^ab^ | 17.45±12.69^a^ | 2.55±1.59^b^ |
| LPL | 33.24±12.52^a^ | 4.81±1.98^b^ | 6.31±3^b^ | 5.36±4.09^b^ | 4.59±1.35^b^ |

Different lowercase letters (a-c) above the bar indicate differences between treatments (*p<0.05*). All results are presented as the mean ± standard deviation (S.D.) (n =3).
